# The Impact of Climate Change and Extreme Weather Conditions on Cardiovascular Health and Acute Cardiovascular Diseases

**DOI:** 10.3390/jcm13030759

**Published:** 2024-01-28

**Authors:** Antonio De Vita, Antonietta Belmusto, Federico Di Perna, Saverio Tremamunno, Giuseppe De Matteis, Francesco Franceschi, Marcello Covino

**Affiliations:** 1 Università Cattolica del Cattolica del Sacro Cuore, 00168 Roma, Italy; antonietta.belmusto@gmail.com (A.B.); federicodiperna1@gmail.com (F.D.P.); francesco.franceschi@unicatt.it (F.F.); macovino@gmail.com (M.C.); 2 Department of Cardiovascular Sciences, Fondazione Policlinico Universitario A. Gemelli, IRCCS, 00168 Roma, Italy; tremamunnosaverio@gmail.com; 3 Department of Internal Medicine and Gastroenterology, Fondazione Policlinico Universitario A. Gemelli, IRCCS, 00168 Roma, Italy; dr.giuseppedematteis@gmail.com; 4 Emergency Medicine, Fondazione Policlinico Universitario A. Gemelli, IRCCS, 00168 Roma, Italy

**Keywords:** climate change, global warming, extreme temperatures, cardiovascular diseases, public health, air pollution

## Abstract

Climate change is widely recognized as one of the most significant challenges facing our planet and human civilization. Human activities such as the burning of fossil fuels, deforestation, and industrial processes release greenhouse gases into the atmosphere, leading to a warming of the Earth’s climate. The relationship between climate change and cardiovascular (CV) health, mediated by air pollution and increased ambient temperatures, is complex and very heterogeneous. The main mechanisms underlying the pathogenesis of CV disease at extreme temperatures involve several regulatory pathways, including temperature-sympathetic reactivity, the cold-activated renin-angiotensin system, dehydration, extreme temperature-induced electrolyte imbalances, and heat stroke-induced systemic inflammatory responses. The interplay of these mechanisms may vary based on individual factors, environmental conditions, and an overall health background. The net outcome is a significant increase in CV mortality and a higher incidence of hypertension, type II diabetes mellitus, acute myocardial infarction (AMI), heart failure, and cardiac arrhythmias. Patients with pre-existing CV disorders may be more vulnerable to the effects of global warming and extreme temperatures. There is an urgent need for a comprehensive intervention that spans from the individual level to a systemic or global approach to effectively address this existential problem. Future programs aimed at reducing CV and environmental burdens should require cross-disciplinary collaboration involving physicians, researchers, public health workers, political scientists, legislators, and national leaders to mitigate the effects of climate change.

## 1. Introduction

Climate change refers to the long-term deviation of temperature trends and weather conditions from seasonal averages. Significant climate shifts recorded since the 19th century might be referred to as increased human activities and fossil fuel combustion, which are responsible for the production and subsequent release into the atmosphere of the so-called greenhouse gases, i.e., carbon dioxide (CO2), methane, nitrogen dioxide (NO2), and ozone. Greenhouse gases play a crucial role in the absorption and re-emission of radiant energy from the sun into the atmosphere, contributing to the greenhouse effect, which is vital for sustaining a habitable range of temperatures on Earth’s surface. In the past two centuries, the excessive production and emission of greenhouse gases and other air pollutants into the atmosphere have been major contributors to climate change and global warming. The rise in atmospheric temperatures has been associated with an increased frequency of natural disasters, such as wildfires, droughts, hurricanes, and coastal flooding. This phenomenon undeniably affects human communities across various fields, including public health [1].

Furthermore, environmental factors have been extensively considered as potential or established risk factors for various acute and chronic health conditions. Accordingly, the “Lancet Commission on Pollution and Health” recommends a multilevel intervention regarding air quality improvement for the prevention and control of non-communicable diseases [2].

Notably, the overall impact of climate change on human health arises not only due to the incidence of extreme weather events; numerous studies have shown a deep connection between climate change and cardiovascular (CV) diseases [3,4,5]. Climate shifts affect the CV system both directly and indirectly by damaging healthcare infrastructure and limiting access to medical assistance, healthcare services and financial resources [6].

In this review, we offer a comprehensive examination of environmental exposures associated with climate change and their intricate relationships. We explore the repercussions of air pollution, extreme temperatures, and various severe weather events on CV disease and try to define the subpopulations particularly vulnerable to CV disease induced by climate change. We also propose strategies to mitigate the CV effects influenced by climate shifts and practical steps that clinicians and healthcare providers should take in their personal lives and as advocates for climate policies. This review, advocating for heightened awareness, introduces, as a novel approach, a groundbreaking healthcare paradigm that embraces the extensive utilization of cutting-edge technologies to address these challenges.

## 2. Epidemiology

Climate change is a global issue, but the amount of exposure to climate shifts, poor air quality, and extreme weather events is widely variable and defines each subject’s vulnerability. Geo-political, socio-economic, and demographic factors are the keys to estimating the health burden all around the world. People living in densely populated areas in developing countries, in the absence of efficient public infrastructure and services, are more vulnerable to climate change-related events. Furthermore, socio-economically disadvantaged subgroups, who experience poor living conditions, more difficult access to healthcare resources, and modern house comforts, represent a vulnerable population [7]. Similarly, the working conditions significantly influence an individual’s susceptibility, especially when engaged in outdoor work or laboring under unsafe conditions, leading to substantial exposure to extreme temperatures and air pollution [8]. Nevertheless, certain non-modifiable factors contribute to an individual’s predisposition to climatic influences. Extreme ages, in particular, confer an elevated susceptibility to climate change-related events, including the impact of extreme temperatures on the CV system [9].

## 3. Air Pollution and Cardiovascular Diseases

Air pollution is defined as a mix of detrimental substances, both particles and gases, released into the atmosphere by human activity, mostly through fossil fuel combustion.

Primary air pollutants are released directly into the atmosphere from either natural sources or human activities. In contrast, secondary pollutants are formed through chemical reactions or physical interactions involving the primary pollutants themselves or other components within the atmosphere. Ozone, carbon monoxide, sulfur dioxide, and nitrogen dioxide are the leading gaseous components. Particulate matter (PM) is further categorized according to size into coarse particles (PM10, <10 μm in diameter), fine particles (PM2.5, <2.5 μm in diameter), and ultrafine particles (<0.1 μm in diameter).

Climate change and air pollution are deeply and mutually connected and share a common source. Increased temperatures are proportionally related to an increasing risk of wildfires and, thus, to an excessive release of air pollutants. Additionally, a warming and dry climate leads to stagnant atmospheric conditions, increasing ground-level ozone, and limited dispersion of air pollutants. Although ozone naturally exists as a molecule in the Earth’s stratosphere, where it serves as a crucial shield by absorbing ultraviolet radiation from the sun, it is known that ground-level ozone differs from stratospheric ozone. At ground level, ozone becomes a significant secondary pollutant, formed through photochemical reactions involving nitrogen oxides and volatile organic compounds, and, acting as a greenhouse gas, contributes to increasing earth temperatures [10].

PM2.5 and NO_2_ are among the most frequently cited pollutants associated with CV risk due to their specific characteristics and the mechanisms through which they affect CV health, such as inhalation and penetration properties, inflammatory responses, vasoconstriction, and endothelial dysfunction.

Inhaling polluted particles is dangerous for health, and the risks vary according to the particles’ size. PM2.5 is the most detrimental air pollutant and plays a pivotal role in contributing to CV disease [11]. Particle deposition in the lungs, mainly PM2.5, stimulates an inflammation cascade, thus resulting in increased blood levels of inflammatory mediators, oxidative stress, and a low-grade systemic inflammatory state [12]. These primary initiating processes activate subsequent effector pathways, such as endothelial dysfunction, pathological changes in vasomotor tone, prothrombotic pathways, autonomic imbalance, hypothalamic-pituitary-adrenal axis activation, and neural reflex arcs, all of which contribute to the development of CV risk factors and subclinical atherosclerosis [13,14,15,16,17].

Furthermore, several studies have demonstrated a relevant association between air pollution and insulin resistance. This relationship may be explained by a PM2.5-induced inflammatory response that widely impairs insulin sensitivity in the liver, adipose tissue, and skeletal muscle [18,19]. Data from a large meta-analysis have also shown that increasing NO2 concentrations and PM2.5 levels in the atmosphere are associated with an increasing incidence and prevalence of type 2 diabetes mellitus [20].

Furthermore, there is compelling evidence indicating a substantial correlation between rising PM2.5 levels and an increase in both systolic and diastolic blood pressure [21,22,23]. Adopting personal strategies to mitigate air pollution exposure, such as the use of face masks and indoor air purifiers, has been demonstrated to significantly lower blood pressure levels [23]. Additionally, chronic exposure to air pollution-induced systemic inflammatory states may influence the burden, progression, and destabilization of atherosclerotic plaques. This, in turn, can contribute to plaque disruption and the occurrence of acute coronary syndrome.

### 3.1. Cardiovascular Mortality

Both short-term and long-term exposure to PM2.5 and other pollutants (i.e., carbon monoxide, NO2, sulfur dioxide, and PM10) increase the risk of CV events [24]. In particular, several previous studies have demonstrated a positive correlation between long-term exposure to PM2.5 and CV mortality in the U.S.A. [25] and Canada [26], despite a very low exposure level to this pollutant. Some prospective cohort studies from China have also demonstrated the same correlation in the setting of high PM2.5 levels [27]. A large meta-analysis has subsequently shown that high exposure to NO2 is associated with increased CV events and CV mortality on the day after exposure [28]. At present, the relationship between ground ozone concentration and CV mortality remains unclear.

### 3.2. Acute Myocardial Infarction

Numerous studies showed that both short-term and long-term exposure to air pollutants increases the incidence of fatal and non-fatal AMI [29,30,31]. The European Study of Cohorts for Air Pollution Effects (ESCAPE project) showed that a 5 μg/m^3^ increase in PM2.5 levels was associated with a 13% increased risk of acute coronary events [29].

Furthermore, short-term exposure to the main air pollutants—except for ozone—has been shown to increase the incidence of AMI [29,30,31].

A multicenter European study showed an association between long-term air exposure—mostly PM2.5 and NO_2_—and the incidence of coronary heart disease and stroke [32].

The pathophysiological basis of this correlation lies in the systemic inflammatory response triggered by inhaling pollutants. This is responsible for the progression and complications of atherosclerotic plaques, thereby amplifying the risk of AMI, especially in patients with a known history of coronary artery disease (CAD) [33,34,35]. Further large-scale studies are required to gain a more comprehensive understanding of this potential association.

### 3.3. Heart Failure

Numerous studies have shown an association between short-term and long-term exposure to air pollutants and heart failure (HF) hospitalization rates and mortality. A prospective cohort study conducted using United Kingdom Biobank data demonstrated a 31% increase in HF incidence due to long-term exposure to air pollutants, after adjustment for confounding variables [36]. Some previous data have shown that PM2.5, PM10, and NO_2_ represent the main pollutants associated with the risk of developing HF and that this relationship may be influenced by genetic polymorphisms and susceptibility [36,37].

### 3.4. Arrhythmias

Cardiac arrhythmias emerge as one of the most clinically relevant CV events resulting from exposure to air pollution. However, the majority of studies assessing the correlation between air pollution and arrhythmias have not investigated specific subtypes of arrhythmias. This lack of specificity is noteworthy, given that the arrhythmia category encompasses a diverse array of clinical conditions with distinct pathophysiological mechanisms.

The main pathogenetic mechanism underlying air pollution-triggered arrhythmias seems to be linked to a low-grade systemic inflammatory state, CV remodeling, and unbalanced cardiac autonomic homeostasis. Inhaled particles depositing inside the lungs trigger an inflammatory response and promote oxidative stress and the release of pro-inflammatory cytokines. A sustained pro-inflammatory response due to chronic exposure to air pollutants enhances both pulmonary and systemic vascular, as well as cardiac, remodeling. Myocardial fibrosis may lead to atrial or ventricular enlargement, providing an organic substrate for different types of arrhythmias (i.e., atrial fibrillation, supraventricular arrhythmias, premature atrial and ventricular complexes, ventricular tachycardias, and ventricular fibrillation).

Furthermore, the presence of a pro-inflammatory environment influences the flow of ions across cell membranes, leading to an extended duration of cardiomyocyte action potential. Nevertheless, air pollution exposure-induced cardiac autonomic dysfunction might also promote the development of brady- and tachy-arrhythmias [38].

The most common type of PM2.5-induced arrhythmia is atrial fibrillation (AF). A cohort study conducted in Korea focused on the pro-arrhythmic effects of long-term exposure to air pollutants and showed that 10 μg/m^3^ increments of PM2.5 were associated with a 17.9% increase in AF incidence [39]. The ARIA study, a multicenter investigation involving patients with implanted devices (i.e., pacemakers or implantable cardioverter-defibrillators), aimed to assess the adverse impact of air pollution on myocardial electrical stability in high-risk patients and revealed a significant relationship between elevated atmospheric PM levels and an increased incidence of ventricular arrhythmias [40].

## 4. Extreme Temperatures and Cardiovascular Disease

Humans, as homeothermic animals, can maintain a constant body temperature. They produce heat through cellular catabolism and muscle contraction, simultaneously retaining it through superficial vasoconstriction. Although there is considerable variability in an individual’s ability to withstand heat stress, some common mechanisms of thermoregulation have been described. These include cooling through the dissipation of heat via lung ventilation (inhaling cool air and exhaling warm air), conduction (through vasodilation and direct contact with a surface), evaporation (through sweating), and radiation [41].

**Figure 1:**
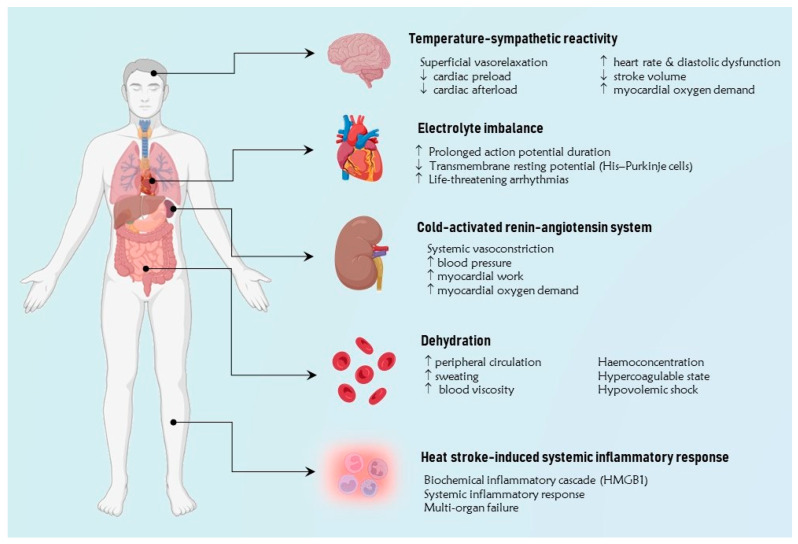
The figure illustrates the main pathophysiological mechanisms implicated in the pathogenesis of cardiovascular disease at extreme temperatures. A simplified approach involves grouping them into five primary categories, including temperature-sympathetic reactivity, cold-activated renin-angiotensin system, dehydration, extreme temperature-induced dysregulation, and heat stroke-induced systemic inflammatory response. HMGB1 = high mobility group protein B1. ↑ Increase, ↓ Reduction.

The main mechanisms underlying the pathogenesis of CV disease at extreme temperatures involve several regulatory pathways, including temperature-sympathetic reactivity, cold-activated renin-angiotensin system (RAS), both cold and heat-mediated dehydration, extreme temperature-induced disionemias, and heat stroke-induced systemic inflammatory response (Figure 1).

### 4.1. Temperature-Sympathetic Reactivity

In the presence of elevated temperatures, the main physiological response is represented by superficial vasorelaxation, which is the dilation of superficial blood vessels, resulting an increase in cutaneous blood flow [42,43]. Furthermore, increasing peripheral circulation redistributes blood in the systemic circulatory system, thus decreasing ventricular preload and systemic vascular resistance (afterload). Accordingly, a higher cardiac output is required to maintain adequate perfusion to all peripheral organs, primarily achieved through a heart rate increase [44,45,46]. The heart rate may indeed rise by an average of 8.5 bpm for each 1 °C increase in body temperature [47]. This hyperdynamic state is often associated with an impairment of diastolic function and a consequent decrease in stroke volume [48]. As CV reserve may be reduced in these conditions, patients with underlying CAD may experience latent or exacerbated symptoms of myocardial ischemia. Conversely, a significant drop in body temperature may trigger an increase in sympathetic response, which results in vasoconstriction of skeletal muscle arteries as well as a catecholamine-driven rise in blood pressure and myocardial oxygen demand [49,50,51]. Interestingly, a few studies have shown that a reduction in body temperature may also cause an increase in the expression of cholesterol crystals in the atherosclerotic plaques, which seriously affects the risk of plaque disruption and AMI in vulnerable subjects [52].

### 4.2. Cold-Activated Renin-Angiotensin System

Some studies have demonstrated that exposure to cold air may cause a significant increase in angiotensin-II plasma levels [53,54]. At low temperatures, synergically with norepinephrine release, renin-angiotensin system (RAS) activation mediates systemic vasoconstriction and arterial hypertension. Sustained high blood pressure also increases myocardial work and oxygen demand, causing a higher risk of CV and cerebrovascular events. Surprisingly, epinephrine serum levels appear unaffected by lower temperatures, suggesting that the increase in norepinephrine within the plasma might not be attributed to adrenal medulla secretion but rather to release from sympathetic nerve endings [55].

### 4.3. Dehydration

Hyperthermia-related vasodilation increases peripheral circulation and sweating, causing significant water loss and subsequent dehydration, which results in haemoconcentration (thrombocytosis and leukocytosis are very common among subjects exposed to extreme heat) [56]. Haemoconcentration also contributes to establishing a hypercoagulable state, which might increase the risk of thrombosis, AMI, and ischemic stroke [57,58]. Severe volume depletion may even evolve into hypovolemic shock [59]. Conversely, a few studies have suggested that hypothermia-related vasoconstriction with reduced peripheral circulation may enhance urinary blood flow and glomerular hyperfiltration, thus promoting water loss and dehydration [60]. Furthermore, lower temperatures may contribute to blood viscosity and favor the precipitation of clotting factors and abnormal proteins (i.e., cold agglutinin), thereby inducing a hypercoagulability status [61]. Therefore, dehydration not only contributes to the CV risk burden through volume depletion alone but may also precipitate coronary and cerebral thrombosis through haemoconcentration and increased blood viscosity. Supporting this relationship between extreme temperatures and dehydration, Lim and colleagues retrospectively analyzed 43.549 patients during 14 years in South Korea and demonstrated how levels of dehydration markers (i.e., blood urea nitrogen-to-creatinine ratio, urine specific gravity, osmolality, and blood hematocrit) described a U-shaped curve as far as environmental temperature linearly changes, with a nadir at the temperature of 22–27 °C. The same curve morphology has been described for the relationship between temperature and CV mortality [62].

### 4.4. Extreme Temperature-Induced Disionemias

Hyperthermia may also cause electrolyte abnormalities (i.e., hypokalemia, hyperkalemia, and hypomagnesemia), which may also increase the risk of cardiac arrhythmias [63,64]. Conversely, hypothermia can induce bradycardia by prolonging the duration of the action potential as well as reducing the transmembrane resting potential of the His–Purkinje cells. Thus, the velocity of impulse conduction may be reduced, triggering life-threatening arrhythmias such as ventricular fibrillation or asystole [64]. Electrocardiogram changes observed in individuals with low body temperatures include prominent J waves, QRS widening, QTc prolongation, and T wave inversion [65,66].

### 4.5. Heat Stroke-Induced Systemic Inflammatory Response

Extreme heat may also trigger conformational changes in the so-called heat-sensitive proteins (i.e., the chaperone family of heat-shock proteins), which activate a biochemical inflammatory cascade involving cytokines and the high mobility group protein B1 (HMGB1), further promoting systemic inflammation and contributing to multi-organ failure [67]. Heat stroke represents a specific nosographic entity characterized as the most serious heat-related illness. It is defined as a hyperthermic state associated with a systemic inflammatory response leading to multi-organ dysfunction. Encephalopathy often emerges as the predominant feature of this disease [68,69], which typically affects the elderly, chronically ill patients, or patients with underlying CV disease whose physiologic cardiac reserve is overwhelmed by heat stress.

## 5. Epidemiology of Temperature-Related Cardiovascular Disease

Extreme temperatures may potentially impact the likelihood of developing diabetes mellitus and worsening glycemic control in patients with preexisting diabetes. Exposure to cold temperatures acts by stimulating the activation of brown adipose tissue, which contributes to thermogenesis through uncoupling mechanisms, thus favoring better glycemic control and insulin sensitivity. Conversely, high mean annual temperatures have been associated with elevated fasting plasma glucose levels, insulin resistance, and an increased incidence and prevalence of diabetes. Short-term temperature variations have also been associated with fluctuations in blood pressure. An inverse relationship between temperature and blood pressure levels has been described in previous studies. A 2017 meta-analysis demonstrated that a decrease in mean outdoor temperature by 1 °C correlated with an increase in systolic blood pressure by 0.26 mmHg and diastolic blood pressure by 0.13 mmHg. This impact was more pronounced in individuals with known CV disease [70]. Furthermore, a few studies showed a significant correlation between increased mean temperatures and rearrangement of the lipid profile, as indicated by decreased levels of plasma high-density lipoprotein (HDL) and increased levels of plasma low-density lipoprotein (LDL) [71]. Moreover, elevated temperatures are linked to reduced time spent engaging in exercise, potentially heightening the long-term risk of CV disease.

### 5.1. Cardiovascular Mortality

There is a significant rise in the relative risk of all-cause mortality and CV mortality when the mean daily temperature exceeds or falls below the optimal range. According to a large previous meta-analysis, a mere 1 °C increase or decrease in mean temperature is linked to a 3.44% and 1.66% increase in CV mortality, respectively [72]. Additionally, a time-series analysis from China revealed a more pronounced impact of cold temperatures on CV mortality compared to elevated temperatures. Indeed, extremely cold temperatures (below the 2.5th percentile) were associated with a notable 92% increase in CV mortality, with sustained effects persisting for more than 14 days, whereas extremely high temperatures (above the 97.5th percentile) were linked to only a 22% increase in CV mortality [73]. Likewise, the Eurowinter Group observed an increase in mortality related to ischemic heart disease and cerebrovascular disease with each 1 °C decrease in temperature among individuals living in Europe [74]. In a recent large, multinational, multicity investigation by Alahmad et al., there was conclusive evidence of a significant increased risk and burden of all-cause CV disease, IHD, stroke, and heart failure mortality from extreme hot and cold temperatures. This study, assessing the associations between extreme temperature and CV cause-specific mortality in 27 countries worldwide, has the merit of including outcomes from countries in different climate zones and with different socioeconomic and demographic characteristics [75].

### 5.2. Acute Myocardial Infarction

Increased AMI incidence is closely associated with both high and low temperatures. The relationship between lower temperatures and AMI has already been extensively described in previous studies. However, the current rising trend in temperatures calls for further studies assessing the impact of global warming on the incidence of acute coronary syndromes.

Interestingly, a 2019 study from Germany showed that during the first years of observation, AMI seemed to be triggered by cold exposure, while the relative risk of heat-related AMI significantly increased at long-term follow-up, especially in patients with diabetes and dyslipidemia [76]. Furthermore, a conclusive meta-analysis of 23 studies showed a significant increase in the relative risk of AMI hospitalization by 1.016 for each 1 °C increase and by 1.014 for each 1 °C decrease in environmental temperature [77].

Interestingly, Guo et al. examined the effects of extreme temperatures on ischemic heart disease (IHD) mortality in five different cities in China. Across these regions, both the relationship between temperature and IHD mortality rates and the temporal relationship between environmental exposure and CV events were very heterogeneous. Overall, IHD mortality increased by about 48% at the 1st percentile of temperature (extremely cold temperatures) compared with the 10th percentile, whereas IHD mortality increased by up to 18% at the 99th percentile of temperature (extremely hot temperatures) compared with the 90th percentile [78].

Bai et al. analyzed 1.4 million hospitalizations for CAD across Ontario between 1996 and 2013, reporting a 9% increase in daily hospitalizations for IHD (95% CI 1% to 16%) and a 29% increase in AMI (95% CI 15% to 45%) on cold days [79].

### 5.3. Heart Failure

Several studies showed a consistent relationship between extreme temperatures and HF admission. A report conducted in South Australia, assessing the potential relation between seasonal variations in environmental temperature and HF morbidity in a cohort of 2961 patients with congestive HF, demonstrated a peak of HF admission and death in Australian winter (July–August), whereas the lowest incidence was in summer (February). Notably, elderly patients were most at risk of seasonal variations in morbidity and mortality. Importantly, during cold months, an increase in respiratory disease (i.e., pneumonia, chronic obstructive pulmonary disease exacerbations) may significantly affect the rate of HF admission [80].

Moreover, a few studies showed a linkage between HF admissions and diurnal temperature range (DTR), expressed as the temperature variability within the day, adjusted for the time trend, seasonality, mean temperature, humidity, and levels of outdoor air pollution [81]. DTR exhibited a significantly greater effect on the HF admission rate in the cooler season in female and elderly patients. Nevertheless, further studies are needed to better characterize the pathogenetic substrate of this correlation between climate change and HF.

### 5.4. Arrhythmias

The heterogeneity of arrhythmia subtypes and their different clinical relevance make the association between environmental temperature and cardiac arrhythmias very difficult to assess. A study conducted on 31,629 arrhythmia-related emergency department admissions in Seoul showed that each 1 °C decrease in mean temperature and each 1 °C increase in diurnal temperature range was associated with an increase in the attributable risk of cardiac arrhythmias (i.e., cardiac arrest, paroxysmal supraventricular tachycardia, atrial fibrillation, and other atrial and ventricular dysrhythmias) by 1.06% and 1.84%, respectively. Women and patients older than 65 years were found to be more susceptible to changes in the diurnal temperature range [82].

A British study reported a temporal correlation between ICD therapy activation and daily outdoor temperatures, as assessed by the nearest temperature monitoring station to the patient’s home address. These data suggested an increased arrhythmic risk at lower but not higher temperatures: the relative risk of ventricular arrhythmias increased by 1.2% for every 1 °C decrease in ambient temperature up to 7 days later and by 11.2% for every 1 °C decrease in temperatures below +2 °C [83].

In a panel of cardiac rehabilitation patients, changes in ambient temperature were associated with decreases in markers of heart rate variability and baroreflex sensitivity, which may lead to an increased risk of arrhythmic events and sudden death in post-infarcted patients [84].

Furthermore, out-of-hospital cardiac arrest (OHCA) has been described as frequently linked to environmental exposure, in particular excessive heat and low humidity. In a US nationwide analysis, exposure to the 90th and 10th percentiles of temperature adjusted to humidity was positively associated with the OHCA with borderline significance. Furthermore, low temperatures during a warm season and high temperatures during a cold season seemed to have a protective effect on OHCA incidence [85].

Other studies from the United States, Germany, and Brazil also revealed an increased risk of ventricular arrhythmias with both extremes of temperature [86,87,88]. Surprisingly, although atrial fibrillation is one of the most prevalent arrhythmias, no study has been planned to evaluate its relationship with ambient temperature.

## 6. Discussion and Future Perspectives

Climate change is modifying global land and ocean temperatures on Earth. Over the last decades, there has been a recorded 1 °C increase in the global mean surface temperature compared to the pre-industrial period, with a significant increase in temperature variability and the occurrence of extreme heat events. Both global warming and extreme temperature exposure are linked to higher CV disease mortality rates, especially in more vulnerable subgroups of patients. Unfortunately, climate change not only alters temperatures but also adversely impacts various environmental factors, including air pollution. Fine PM is strictly linked to CV disease morbidity and mortality. Studies have compellingly illustrated the compounded and severe impact of concurrent exposure to air pollution and heat on CV disease mortality [13,75].

**Figure 2:**
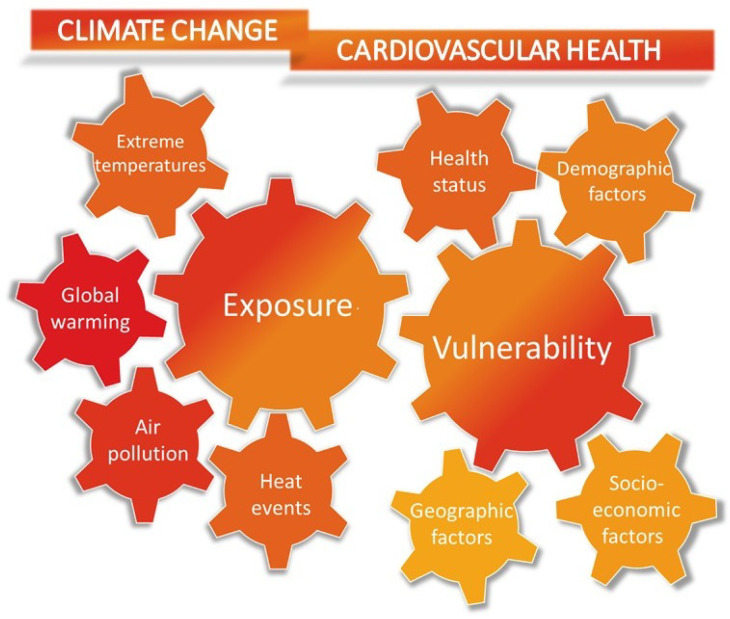
The figure illustrates the main factors contributing to the cardiovascular risk of climate change. The increased incidence of extreme heat events due to global warming and air pollution is significantly correlated with the rate of cardiovascular mortality and cardiovascular disease. Individuals with a higher vulnerability to heat-related acute cardiovascular events, such as myocardial infarction, stroke, acute heart failure, and arrhythmias, include older individuals, those with lower socioeconomic status, and individuals with underlying clinical conditions like type 2 diabetes mellitus and hypertension.

Importantly, the impact of climate change on CV health varies across demographic and socioeconomic subgroups residing in different geographical areas. Regions with coastal and low-lying geography, as well as densely populated cities lacking proper infrastructure, are less shielded from potential health risks associated with extreme climate-related events. Additional socio-economic factors, such as homelessness, housing type, and insufficient green spaces, contribute to this vulnerability. Age and previous CV disease configure a subpopulation of patients at increased risk of climate-related CV events and death. Refugees and immigrants represent another subgroup with an increased risk of CV events associated with climate change. Factors such as language barriers, substandard living and working conditions, and socioeconomic disparities have been associated with heightened vulnerability to adverse CV outcomes [13,41] (Figure 2). 

Climate change and CV health mutually influence each other and configure socioeconomic burdens, sharing potential preventive policies. Untargeted strategies or suggestions for individual-level intervention are unlikely to be the most efficient approach in terms of cost, effort, or equity for both climatic changes and CV disease. There is an urgent need for a comprehensive intervention that spans from the individual level to a systemic or global approach to effectively address this existential problem.

Future programs aimed at reducing CV and environmental burdens should require cross-disciplinary collaboration involving physicians, researchers, public health workers, political scientists, legislators, and national leaders to mitigate the effects of climate change [89].

One hundred ninety countries signed the Paris Agreement in 2015 [90], engaging to restrict global warming to less than 2.0 °C above preindustrial levels. Some previous analyses have predicted the prevention of significant increases in temperature-related CV mortality by adhering to the temperature targets set by the Paris Agreement. However, to establish effective measures, policies addressing the link between climate change and CV diseases may need implementation across multiple levels and require the involvement of various stakeholders. Importantly, evaluating patient-specific and community health risks, along with the extent of climate-related exposures, is crucial for implementing any improvement strategy.

In the matter of CV disease prevention, physicians often give patients advice regarding lifestyle, including diet, physical activity, and avoiding cigarette smoking, as well as checking blood pressure and glucose and cholesterol serum levels. Since climate change has been established as a potential risk factor for CV diseases, physicians should also inform patients about it. As a result, recommendations should include measures such as preventing dehydration and limiting exposure to extreme temperatures. Additionally, individuals should use face masks and take precautions to shield themselves from high levels of air pollutants.

Furthermore, given the heightened risk of mortality during heat waves for patients with CV diseases, it is advisable for clinicians to offer practical guidance aimed at helping patients manage heat exposure. Evidence-supported advice includes: (1) increasing fluid intake; (2) staying in a cool or air-conditioned environment and wearing loose-fitting clothes; (3) reducing normal activity levels; and (4) providing patients with customized “heatwave rules” specific to their CV medications. For instance, this may involve careful home monitoring of weight, blood pressure, and symptoms, as well as adjusting diuretic or antihypertensive doses on particularly hot days [91,92,93].

As a bidirectional relationship exists between climate change and CV health, climate change mitigation and adaptation strategies, particularly those involving exercise and diet, may yield specific CV health co-benefits.

It is noticeable, indeed, that practicing regular aerobic physical activity (i.e., walking, jogging, and cycling) represents one of the most relevant strategies to reduce the risk of CV diseases and improve the management of traditional CV risk factors. Moreover, the evolving active transport strategies represent one of the most sustainable modes of personal transportation, as they do not elevate the CO2 emissions rate and, consequently, do not contribute to climate change [94]. However, practicing outdoor physical activity in regions with poor air conditions due to air pollution might reduce its protective effects on CV health and CV risk burden, thus explaining how climate change and air pollution might indirectly affect human health and CV disease prevention [41]. Likewise, extreme temperatures and heat waves may limit the quality, duration, and effort tolerance of physical exercise, thus lowering its beneficial effect on the CV system.

Aequilibrated dietetic habits play an important role in reducing CV disease risk and progression. Mediterranean-type diets, which are rich in fruit and vegetables and poor in animal proteins, were revealed to be healthier and have a positive impact on CV health and risk factors [95]. Furthermore, promoting a reduction in meat consumption, especially from methane-producing animals (i.e., sheep and cows), is also associated with a decrease in overall greenhouse gas production and an improvement in air quality [96].

Certainly, assessing CV medications in the context of health and climate change is an important consideration. CV therapy could influence a patient’s susceptibility to heat-related conditions, such as dehydration and electrolyte imbalance. The individual response to medical treatment may significantly differ based on various environmental settings and temperatures. Given the different ranges of patients’ reactions to medications and environmental factors, personalized care plans are essential. For example, the dose of antihypertensive drugs should be adjusted according to environmental temperatures to reduce the heat-induced risk of hypovolemia, hypotension, and syncope [97]. Beta-blockers decrease blood flow to the skin and reduce cardiac output, thereby increasing the susceptibility of individuals to heatstroke. Statins may offer thermal protection primarily through the promotion of cutaneous vasodilation. Preclinical studies have also indicated that statins may mitigate the inflammatory response triggered by exposure to air pollution. Recognizing how medications influence sensitivity to heat and pollutants might allow clinicians to provide personalized recommendations regarding their patients’ risks in cases of extreme exposure. Thus, evaluating CV medications in light of climate change and heat-related illnesses requires assessing the risks related to various medications, closely monitoring patients, and implementing tailored care plans to secure optimal health outcomes through evolving environmental conditions.

As a conclusion, increasing awareness among healthcare professionals regarding the health impact of climate change is crucial to diminishing the so-called “residual CV risk” that goes beyond conventional CV risk factors. Overcoming the primary obstacle of tailoring preventive strategies involves a comprehensive assessment and continual reassessment of individual vulnerability to environmental exposures. In this perspective, this review introduces, as a novel approach, a groundbreaking healthcare paradigm that embraces the extensive utilization of cutting-edge technologies to address these challenges. Leveraging real-time data on air pollutant concentrations, temperature, and humidity levels through dedicated apps or web platforms may facilitate prompt administration of therapeutic and preventive advice to individuals at risk. Integration of such data with the growing pool of self-assessed and clinically detected parameters from telehealth programs is essential. Furthermore, developing artificial intelligence and machine learning software might support clinicians by providing prediction models of exposure and subsequent recommendations for preventive measures and medications.

## 7. Conclusions

Climate change-related CV burden represents one of the latest challenges in preventive cardiology that physicians are facing. Although the impact of climate change on human health remains to be accurately estimated, several studies have demonstrated its negative effects on the risk and progression of CV diseases. Therefore, serious strategies should be planned to contrast global warming and climate change, involving physicians, researchers, public health professionals, political scientists, legislators, and national leaders who have to work together to alleviate the impacts of climate change and preserve public health.

## Data Availability

Not applicable.
